# Ravulizumab pharmacokinetics and pharmacodynamics in patients with generalized myasthenia gravis

**DOI:** 10.1007/s00415-023-11617-1

**Published:** 2023-03-09

**Authors:** Tuan Vu, Stephan Ortiz, Masahisa Katsuno, Djillali Annane, Renato Mantegazza, Kathleen N. Beasley, Rasha Aguzzi, James F. Howard

**Affiliations:** 1 University of South Florida Morsani College of Medicine, Tampa, FL USA; 2 Alexion, AstraZeneca Rare Disease, Boston, MA USA; 3 Nagoya University Graduate School of Medicine, Nagoya, Japan; 4 Hôpital Raymond Poincaré, University of Versailles, Garches, France; 5 Fondazione IRCCS Istituto Neurologico Carlo Besta, Milan, Italy; 6 The University of North Carolina, Chapel Hill, NC USA

**Keywords:** Complement, Generalized myasthenia gravis, Pharmacodynamics, Pharmacokinetics, Ravulizumab

## Abstract

**Introduction:**

The terminal complement C5 inhibitor ravulizumab has a long elimination half-life, allowing maintenance dosing every 8 weeks. In the 26-week, double-blind, randomized, placebo-controlled period (RCP) of the CHAMPION MG study, ravulizumab provided rapid and sustained efficacy and was well tolerated in adults with anti-acetylcholine receptor antibody-positive (AChR Ab+) generalized myasthenia gravis (gMG). This analysis evaluated the pharmacokinetics (PK), pharmacodynamics (PD), and potential immunogenicity of ravulizumab in adults with AChR Ab+ gMG.

**Methods:**

Data were analyzed from 86 patients who received ravulizumab in the CHAMPION MG RCP. Ravulizumab dosing was weight-based: initial loading dose of 2400/2700/3000 mg on Day 1 and maintenance doses of 3000/3300/3600 mg on Day 15 and then every 8 weeks. PK parameters were estimated from serum ravulizumab concentrations determined pre- and post-dose; PD effects of ravulizumab on serum free C5 concentrations were measured; and immunogenicity was assessed using anti-drug antibody and neutralizing-antibody assays.

**Results:**

Target serum ravulizumab concentrations (> 175 µg/mL) were achieved immediately after the first ravulizumab dose (within 30 min of infusion completion) and maintained throughout the 26-week treatment period irrespective of patient body weight. Following the final maintenance dose, mean *C*_max_ was 1548 µg/mL and *C*_trough_ 587 µg/mL; no meaningful differences were noted among body-weight categories. Inhibition of serum free C5 was immediate, complete (< 0.5 μg/mL), and sustained throughout treatment in all patients. No treatment-emergent anti-drug antibodies were observed.

**Conclusions:**

PK/PD evidence supports the use of ravulizumab every 8 weeks for immediate, complete, and sustained inhibition of terminal complement C5 in adults with AChR Ab+ gMG.

**Trial registration:**

ClinicalTrials.gov ID: NCT03920293 (April 18, 2019).

**Supplementary Information:**

The online version contains supplementary material available at 10.1007/s00415-023-11617-1.

## Introduction

Myasthenia gravis (MG) is a rare, chronic, debilitating autoimmune disease arising from impaired neuromuscular transmission [1–4]. MG is characterized by fluctuating muscle weakness and by exertional and potentially disabling fatigability [1, 5]. It is a heterogeneous condition that may cause localized (most commonly ocular) muscle weakness, but is more frequently generalized (gMG), presenting with differing levels of weakness in muscle groups of the head, neck, trunk, and/or limbs [1, 4]. The symptoms of gMG can substantially impact a patient’s daily personal and social functioning, work activities, economic well-being, and quality of life [1, 6–14].

The majority of patients (~ 85%) with gMG have autoantibodies directed against the postsynaptic acetylcholine receptor (AChR); binding of these autoantibodies has been shown to activate the classical complement cascade [2, 3, 15–18]. This leads to the formation of terminal complement protein C5, which is cleaved into C5a and C5b [2, 15]. C5b combines with other complement proteins (C6, C7, C8, and multiple C9 molecules) to form the terminal membrane attack complex (MAC; also known as the terminal complement complex), which causes architectural destruction of the neuromuscular junction (NMJ) and hence impaired neuromuscular transmission, leading to muscle weakness [1, 2, 4, 15]. Inhibition of terminal complement activation is, therefore, a rational approach to prevent formation of the MAC—and, presumably, destruction of the NMJ—in patients with anti-AChR antibody-positive (AChR Ab+) gMG [19].

Ravulizumab is a humanized monoclonal antibody that specifically binds with high affinity to the human terminal complement protein C5 [20], inhibiting its activation by blocking its cleavage and thus preventing the cascade of events that leads to MAC-mediated destruction of the NMJ and consequent muscle weakness. Ravulizumab was engineered from the C5 complement inhibitor eculizumab by incorporating specific amino acid substitutions that result in more efficient dissociation from C5 following endocytosis of the bound antibody, and enhanced neonatal Fc receptor-mediated recycling of the unbound antibody, thus extending the molecule’s elimination half-life and its duration of action [20, 21]. Due to this prolonged half-life, ravulizumab is associated with a low burden of administration (dosing every 8 weeks) and delivers sustained therapeutic serum concentrations suited to addressing fluctuations in muscle weakness and limiting the risk of exacerbations and crises [22].

Phase 3 studies in patients with complement-associated disorders (paroxysmal nocturnal hemoglobinuria [23, 24] and atypical hemolytic uremic syndrome [25]) showed that ravulizumab administered every 8 weeks provided immediate, complete, and sustained terminal complement C5 inhibition, and was well tolerated. Over 26 weeks of treatment, ravulizumab administered every 8 weeks was non-inferior to eculizumab administered every 2 weeks in patients with paroxysmal nocturnal hemoglobinuria who were clinically stable on previous eculizumab therapy [24]. Results from a further 26 weeks of treatment with ravulizumab in the same study showed that patients experienced durable efficacy [26]. Efficacy was consistent in those who switched from eculizumab to ravulizumab after the first 26 weeks and, importantly, all patients, including those with serum free C5 levels ≥ 0.5 μg/mL while receiving eculizumab, showed complete terminal complement inhibition (serum free C5 < 0.5 μg/mL) after switching to ravulizumab, indicating improved serum free C5 control [26].

Most recently, ravulizumab has demonstrated rapid, sustained efficacy and good tolerability in patients with AChR Ab+ gMG in the randomized, placebo-controlled period (RCP) of the phase 3 CHAMPION MG study [27], and was approved by the US Food and Drug Administration in April 2022 for the treatment of adults with AChR Ab+ gMG [22]. Ravulizumab was approved in Japan in August 2022 for the treatment of adults with AChR Ab+ gMG whose symptoms are difficult to control with high-dose intravenous immunoglobulin therapy (IVIg) or plasmapheresis, and in the European Union in September 2022 as an add-on to standard therapy for the treatment of adults with AChR Ab+ gMG. The present investigation used data and samples from the CHAMPION MG study to evaluate the pharmacokinetics, pharmacodynamics, and potential immunogenicity of ravulizumab (maintenance dosing every 8 weeks) in adults with AChR Ab+ gMG.

## Methods

### Study population and study design

This analysis was based on pharmacokinetic, pharmacodynamic, and immunogenicity data from the double-blind RCP of the phase 3, multinational CHAMPION MG study of ravulizumab in adults with AChR Ab+ gMG (ClinicalTrials.gov ID: NCT03920293). Details of the study design and clinical efficacy and safety results have been reported elsewhere [27].

In brief, the CHAMPION MG study enrolled 175 patients aged ≥ 18 years who had a diagnosis of AChR Ab+ MG ≥ 6 months before screening, a Myasthenia Gravis–Activities of Daily Living (MG-ADL) total score of ≥ 6 at screening and randomization, and no previous treatment with a complement inhibitor. A total of 86 patients received ravulizumab for 26 weeks and 89 patients received placebo. Intravenous dosing of ravulizumab was based on the patient’s body weight [23, 25], with an initial loading dose of 2400 mg (body weight ≥ 40 to < 60 kg), 2700 mg (body weight ≥ 60 to < 100 kg), or 3000 mg (body weight ≥ 100 kg) at baseline (Day 1), and maintenance doses of 3000, 3300, or 3600 mg, respectively, on Day 15 (Week 2) and then every 8 weeks. Use of plasma exchange/plasmapheresis (PLEX/PP) or IVIg was permitted for patients who required rescue treatment following clinical deterioration. For patients who received PLEX/PP, a supplemental body weight-based dose of ravulizumab was administered within 4 h after completing each PLEX/PP intervention. For a PLEX/PP intervention during the loading phase (from the administration of the loading dose until the first maintenance dose), patients were administered supplemental doses of either 1200 mg (body weight ≥ 40 to < 60 kg) or 1500 mg (≥ 60 kg) ravulizumab; for an intervention in the maintenance dose phase (from the first maintenance dose onwards), patients were prescribed supplemental doses of either 1500 mg (≥ 40 to < 100 kg) or 1800 mg (≥ 100 kg) ravulizumab. For patients who received IVIg, for all body weight groups, a supplemental ravulizumab dose of 600 mg was administered within 4 h after completing the last continuous IVIg session. Stable doses of immunosuppressant therapies (including oral corticosteroids) and cholinesterase inhibitors were permitted throughout the RCP. All participants gave their informed consent prior to their inclusion in the study.

The current analysis of pharmacokinetic/pharmacodynamic data was performed on the 86 patients who received ravulizumab in the RCP of the CHAMPION MG study.

### Sample collection and analyses

Blood samples to measure baseline and trough levels of serum ravulizumab, serum free C5 (pharmacodynamics), and anti-drug antibody (ADA) evaluation were collected pre-dose (within 30 min before the start of infusion of study drug). Blood samples for peak post-treatment serum pharmacokinetic and pharmacodynamic assessments were taken within 30 min after completion of study drug infusion. Blood samples were obtained at baseline and at Weeks 2, 10, 18, and 26. Samples were refrigerated (2–8 °C [36–46 °F]) after processing and shipped on ice to the analysis site.

#### Ravulizumab pharmacokinetics

Total (bound plus free) serum concentrations of ravulizumab were quantified using validated liquid chromatography with tandem mass spectrometry, with sensitivity (lower limit of quantification) of 1.00 µg/mL and precision (coefficient of variation) of ≤ 13.8% (Alexion, AstraZeneca Rare Disease, data on file).

#### Free C5 concentration

Concentrations of free C5 in serum (as a measure of target engagement) were measured using a validated microfluidics assay with fluorescence detection. The assay has sensitivity (lower limit of quantification) of 0.0183 µg/mL and precision (coefficient of variation) of ≤ 15.6% (Alexion, AstraZeneca Rare Disease, data on file).

#### Immunogenicity

Serum concentrations of ADAs against the drug were measured using a validated electrochemiluminescence ligand binding assay with sensitivity of 80.7 ng/mL and precision (coefficient of variation) of ≤ 16.1%. Samples proving positive in the ADA assay were further characterized for assessments of ADA titer and the presence of neutralizing antibodies.

### Pharmacokinetic, pharmacodynamic, and immunogenicity endpoints

Pre-specified pharmacokinetic endpoints were the change in serum ravulizumab concentration over time, including the maximum/peak observed serum concentration (*C*_max_) and the serum concentration at the end of the dosing interval (*C*_trough_).

The pharmacodynamic effects of ravulizumab were measured as absolute values and changes and percentage changes from baseline in serum free C5 concentration over time. In these analyses, complete terminal complement inhibition was defined as a serum free C5 concentration of < 0.5 μg/mL (Alexion, AstraZeneca Rare Disease, data on file).

Immunogenicity was assessed as the incidence of treatment-emergent ADAs throughout the duration of the study. Patients with treatment-emergent ADA responses would be further assessed for the impact of immunogenicity on pharmacokinetics and pharmacodynamics.

### Statistical analyses

Pharmacodynamic and immunogenicity (ADA) outcomes were assessed in the full analysis set and the safety analysis set, respectively, both of which included all randomized patients who received at least one dose of ravulizumab. The pharmacokinetics analysis set included all treated patients with at least one post-baseline pharmacokinetic concentration available.

Pharmacokinetic parameters (estimated using non-compartmental analyses) and absolute values and changes from baseline in free C5 serum concentrations are summarized using descriptive statistics (Phoenix™ Pharmacokinetic and Pharmacodynamic Platform Version 8.3; Certara, Princeton, NJ, USA). The incidence of ADAs and neutralizing antibodies was analyzed by summarizing the number and percentage of patients who developed detectable ADAs or neutralizing antibodies in the ravulizumab-treated population.

## Results

### Patients

All 86 patients randomized to receive ravulizumab were eligible for inclusion in the safety analysis (immunogenicity), full analysis (pharmacodynamics), and pharmacokinetics analysis sets. Patient baseline characteristics have been described previously [27]. In summary, approximately half of the patients were female (44 patients; 51%); 67 patients (78%) were White, 15 (17%) were Asian (six from Japan), two (2%) were Black/African American, and race was unreported for two patients. Mean (standard deviation [SD]) characteristics at baseline were: age at first study dose of ravulizumab 58.0 (13.8) years; body weight 91.6 (23.4) kg (range 40.0–165.8 kg). Patients had a mean (SD) MG-ADL total score of 9.1 (2.6) and a mean Quantitative Myasthenia Gravis total score of 14.8 (5.2) at baseline. During the RCP of the study, 85 patients (99%) who were administered ravulizumab received concomitant MG medications: 72 (84%) received acetylcholinesterase inhibitors, 57 (66%) received systemic corticosteroids, and 56 (65%) received non-steroidal immunosuppressants.

### Pharmacokinetics

**Fig. 1:**
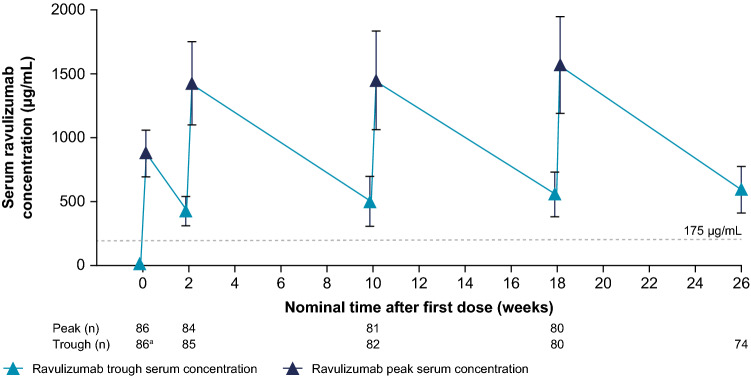
Mean (SD) concentration–time profile of ravulizumab over 26 weeks. Target serum concentrations of ravulizumab (> 175 µg/mL) were achieved immediately after the first dose and sustained throughout the entire 26-week treatment period. ^a^Baseline. Ravulizumab *C*_trough_ and *C*_max_ were measured in blood samples collected within 30 min before the start of ravulizumab infusion and within 30 min after the completion of ravulizumab infusion, respectively. *C*_max_ maximum observed serum concentration, *C*_trough_ serum concentration at the end of the dosing interval, *SD* standard deviation

**Fig. 2:**
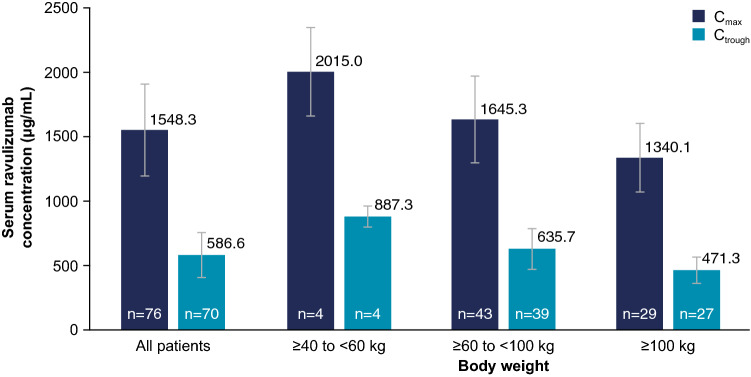
Mean (SD) ravulizumab *C*_max_ and *C*_trough_ serum levels at last assessment (Weeks 18 and 26, respectively) by patient body weight. No notable differences in ravulizumab *C*_max_ or *C*_trough_ were observed among weight categories. Excludes pharmacokinetic data collected after supplemental dose administration following clinical deterioration. *C*_*max*_ maximum observed serum concentration, *C*_*trough*_ serum concentration at the end of the dosing interval, *SD* standard deviation

Target serum concentrations of ravulizumab (> 175 µg/mL) [28] were achieved immediately after the first dose (within 30 min of completion of infusion) and sustained in all patients at all time points assessed throughout the 26-week treatment period (Fig. 1). Subsequent to the first (loading) dose, mean (SD) ravulizumab *C*_max_ and *C*_trough_ were 874 (184) µg/mL and 418 (116) µg/mL, respectively. Following the final maintenance dose, mean *C*_max_ and *C*_trough_ were 1548 (359) µg/mL and 587 (174) µg/mL, respectively. No notable differences were observed among weight categories with this weight-based ravulizumab dosing regimen (Fig. 2).

#### Supplemental dosing

Two patients received PLEX/PP interventions during the maintenance treatment phase of the study (one patient received four PLEX/PP interventions, and the other received two); both received subsequent weight-based supplemental ravulizumab doses of 1800 mg within 4 h after the end of each PLEX/PP intervention. For each instance of PLEX/PP, the prescribed supplemental dose successfully offset the marked decline in serum ravulizumab concentration, while not exceeding the maximum observed ravulizumab concentrations observed for these patients at other points in the study. Five patients received IVIg interventions during the maintenance treatment phase (one patient received three courses of IVIg on separate occasions, and the other four patients each received one course of IVIg). All five patients were administered supplemental ravulizumab doses of 600 mg within 4 h after completing the last IVIg infusion of the course, which offset the increased clearance of ravulizumab following IVIg rescue treatment and maintained ravulizumab concentrations above the target threshold of 175 μg/mL.

The impact of PLEX/PP and IVIg interventions and supplemental ravulizumab dosing on the serum concentration–time profile of ravulizumab is shown in representative examples in Supplementary Figs. S1 and S2, respectively.

### Pharmacodynamics

**Fig. 3:**
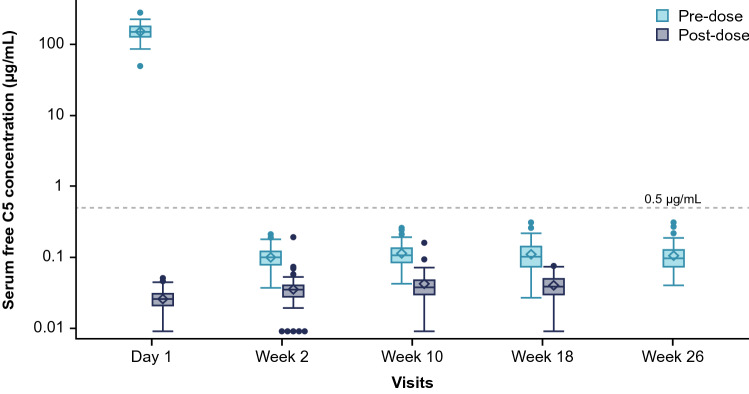
Serum-free C5 concentration box plot across 26 weeks of treatment with ravulizumab. Complete terminal complement inhibition (serum free C5 < 0.5 µg/mL) was sustained throughout 26 weeks of treatment in all patients at all time points. For serum free C5 values that were below the limit of quantification, the lower limit of quantification/2 = 0.00915 μg/mL was utilized. The Y-axis is presented on a log scale. The horizontal line in the middle of each box indicates the median, the diamond indicates the mean, and the top and the bottom borders of the box mark the 75th and 25th percentiles, respectively. The whiskers represent the 1.5 interquartile range of the lower and upper quartiles. Outliers are represented by circles beyond the whiskers. The dashed horizontal line indicates a serum free C5 concentration of 0.5 μg/mL, levels below which indicate complete inhibition. *C5* complement component 5

At baseline (before the first dose), the mean (SD) serum free C5 concentration was 153.6 (37.0) µg/mL, with a range of 50.4–286.0 µg/mL. Administration of ravulizumab achieved immediate and complete terminal complement inhibition (defined as serum free C5 < 0.5 μg/mL), as observed by the end of the first ravulizumab infusion. Complete inhibition was sustained throughout 26 weeks of treatment in all patients at all time points assessed (Fig. 3). A combined plot showing the serum C5 concentration and mean MG-ADL total scores over time is shown in Supplementary Fig. S3.

#### Supplemental dosing

Serum-free C5 concentrations in samples that were collected before or after supplemental ravulizumab dosing following PLEX/PP or IVIg interventions were maintained below 0.5 µg/mL.

### Immunogenicity

No treatment-emergent ADAs were observed in any of the patients receiving ravulizumab during 26 weeks of treatment. Eight (9.3%) patients were ADA-positive at baseline (pre-treatment) but were ADA-negative at all times following the first dose of ravulizumab. The ADA titers were low and transient, no apparent impact of immunogenicity on ravulizumab pharmacokinetics/pharmacodynamics was observed, and all assay results for anti-drug neutralizing antibodies in ADA-positive samples were negative.

## Discussion

The RCP of the CHAMPION MG phase 3 trial demonstrated the efficacy and well-tolerated profile of ravulizumab administered every 8 weeks in adult patients with AChR Ab+ gMG [27]. The current analysis of pharmacokinetic, pharmacodynamic, and immunogenicity data provides evidence to confirm the dosing regimen for intravenous ravulizumab in adult patients with AChR Ab+ gMG.

For many of the treatments used in patients with AChR Ab+ gMG, there are limited data on the speed of onset of effect. The present analysis of data from the CHAMPION MG study shows that ravulizumab weight-based dosing in patients with AChR Ab+ gMG achieves target serum concentrations immediately after the first dose (within 30 min of the end of infusion). Furthermore, these concentrations were sustained, irrespective of patient body weight (ranging from 40 to 166 kg), through 26 weeks of treatment (maintenance dose administered every 8 weeks). The weight-based dosing regimen reduced exposure differences across the adult body weight range. The pharmacokinetic findings are consistent with ravulizumab’s mean elimination half-life of 56.6 days (8.1 weeks) [22], which is substantially longer than that of the terminal C5 inhibitor eculizumab (18.2 days) in patients with AChR Ab+ gMG [29]. Ravulizumab’s longer half-life, allowing dosing every 8 weeks, has the potential for easing patient burden with less frequent administration in comparison to eculizumab.

The pharmacodynamic analyses presented here support ravulizumab’s mechanism of action, demonstrating that it rapidly blocks serum free C5, achieving complete inhibition of terminal complement (serum free C5 < 0.5 μg/mL) by the end of the initial infusion and sustaining this inhibition through the 26-week treatment period. In a previous pharmacodynamic analysis, eculizumab achieved complete inhibition of terminal complement at all time points in 92% of patients with gMG treated with the drug in a clinical study [29]; in the CHAMPION MG study, however, ravulizumab completely inhibited terminal complement in all patients at all time points. The consistent long-term suppression of complement activation by ravulizumab is particularly beneficial in gMG as it is a chronic, fluctuating disease that is prone to unpredictable exacerbations and crises if inadequately controlled. No treatment-emergent ADAs were observed in any of the patients treated with ravulizumab during 26 weeks of treatment. The baseline ADA positivity observed in a few complement-naive patients is most likely due to the presence of antibodies in response to environmental exposure to non-human proteins, glycans, or structurally similar products [30, 31]. This is consistent with findings across the approved indications for ravulizumab: in 449 patients treated with ravulizumab in clinical trials in paroxysmal nocturnal hemoglobinuria, atypical hemolytic uremic syndrome, and gMG, only two cases (0.45% of patients) of treatment-emergent ADAs have been reported (in one adult with paroxysmal nocturnal hemoglobinuria and one adult with atypical hemolytic uremic syndrome) [32]. In both cases, the ADAs were of low titer, transient, and did not correlate with clinical response or adverse events.

PLEX/PP and IVIg have been shown to reduce serum ravulizumab concentrations and a supplemental dose of ravulizumab is required to compensate for these interventions [22]. Although data in the current study are limited, there was no accumulation of ravulizumab with the use of the supplemental dose indicated in the prescribing information, and ravulizumab concentrations were maintained above the therapeutic threshold for the duration of the dosing interval. Although no interaction with commonly administered MG medications was anticipated, it was notable that despite a high proportion of patients receiving concomitant medications for MG, there were no unexpected findings with respect to either serum ravulizumab or serum free C5 concentrations and all patients achieved serum free C5 concentrations below the target threshold. As ravulizumab is a therapeutic monoclonal antibody and, therefore, a relatively large molecule, there is a low likelihood of drug–drug interaction via mechanisms such as CYP450, or P-glycoprotein or other transporters.

These pharmacokinetic and pharmacodynamic findings—rapid achievement of therapeutic ravulizumab concentrations and rapid inhibition of serum free C5 concentrations that were sustained through the 26-week treatment period—support the clinical results of the CHAMPION MG study, which showed the treatment effect of ravulizumab appeared within the first week following infusion and was sustained throughout 26 weeks [27]. Clinical improvements were observed in measures of activities of daily living (MG-ADL) and disease severity (Quantitative Myasthenia Gravis score), and ravulizumab was well tolerated with a side-effect profile generally comparable with that of placebo [27]. There were no cases of meningococcal infection during the RCP of CHAMPION MG. One case of meningitis has been reported during the ongoing open-label extension; however, the causative organism was not identified as *Neisseria meningitidis* [33]. The risk of meningococcal infection that arises from ravulizumab’s mechanism of action is mitigated by the requirement for patients to be vaccinated against meningococcal infections at least 2 weeks before initiating ravulizumab treatment [22].

It is interesting to speculate why the clinical response to complement inhibition is so rapid, given that complement activation putatively destroys the NMJ postsynaptic AChR and/or membrane [2, 15]. One possible explanation is the dynamic nature of the postsynaptic AChR: it is being continually degraded and replaced in the course of normal physiology, as shown by in vivo imaging studies in mice [34, 35]. Similar studies also indicate the half-life of membrane AChRs is approximately 9–14 days [35–37]; thus, if the ongoing destruction of the NMJ via the MAC is prevented by inhibiting complement activation, the postsynaptic AChR density can theoretically recover within the timeframe of the observed clinical response. This is also consistent with the observation that a rapid clinical response follows the removal of pathogenic antibodies via PLEX or Fc receptor inhibition [1].

Overall, ravulizumab appears well placed to offer a sustained treatment effect, without the burdensome side effects associated with long-term immunosuppressive therapies [38, 39]. In addition, the rapid effect of ravulizumab in directly targeting the terminal complement system favorably contrasts with the slower onset of action of non-steroidal immunosuppressant therapies, most of which generally take several months to become effective [40]. In practice, this predictable, rapid effect enables patients to regain function quickly following ravulizumab infusion [27]. As gMG is a chronic disease with an ongoing treatment burden, the relative convenience and predictability of ravulizumab dosing every 8 weeks may additionally benefit patients’ daily lives, potentially easing the treatment burden and improving treatment accessibility.

## Conclusions

The results of this study provide pharmacokinetic and pharmacodynamic evidence to support the use of ravulizumab every 8 weeks for immediate (within 30 min of the end of the first infusion), complete, and sustained inhibition of terminal complement in patients with AChR Ab+ gMG.

## Supplementary Information

Below is the link to the electronic supplementary material.

Supplementary file1 (DOCX 375 KB)

## Data Availability

Alexion, AstraZeneca Rare Disease will consider requests for disclosure of clinical study participant-level data provided that participant privacy is assured through methods like data de-identification, pseudonymization, or anonymization (as required by applicable law), and if such disclosure was included in the relevant study informed consent form or similar documentation. Qualified academic investigators may request participant-level clinical data and supporting documents (statistical analysis plan and protocol) pertaining to Alexion-sponsored studies. Further details regarding data availability and instructions for requesting information are available in the Alexion Clinical Trials Disclosure and Transparency Policy at https://alexionclinicaltrials.com/Disclosure-and-Transparency-Policy. Link to Data Request Form: https://alexion.com/contact-alexion/medical-information.
