# Supplementary material for: Ravulizumab pharmacokinetics and pharmacodynamics in patients with generalized myasthenia gravis
Source: J Neurol. 2023 Mar 9;270(6):3129–37. doi: 10.1007/s00415-023-11617-1 (PMC10188401; doi:10.1007/s00415-023-11617-1)
Supplement: Supplementary file 1 — Supplementary file1 (DOCX 375 KB) [file 415_2023_11617_MOESM1_ESM.docx]

***Supplementary material***

# Ravulizumab pharmacokinetics and pharmacodynamics in patients with generalized myasthenia gravis

Tuan Vu,^1^ Stephan Ortiz,^2^ Masahisa Katsuno,^3^ Djillali Annane,^4^ Renato Mantegazza,^5^ Kathleen N Beasley,^2^ Rasha Aguzzi,^2^ James F Howard, Jr^6^

^1^*University of South Florida Morsani College of Medicine, Tampa, FL, USA;* ^2^*Alexion, AstraZeneca Rare Disease, Boston, MA, USA;* ^3^*Nagoya University Graduate School of Medicine, Nagoya, Japan;* ^4^*Hôpital Raymond Poincaré, University of Versailles, Garches, France;* ^5^*Fondazione IRCCS Istituto Neurologico Carlo Besta, Milan, Italy;* ^6^*The University of North Carolina, Chapel Hill, NC, USA*

**Corresponding author**
Stephan Ortiz; Email: [Stephan.Ortiz@alexion.com](mailto:Stephan.Ortiz@alexion.com)

**Supplementary Fig. S1** Example of impact of PLEX/PP intervention and supplemental ravulizumab doses on the concentration–time profile of ravulizumab


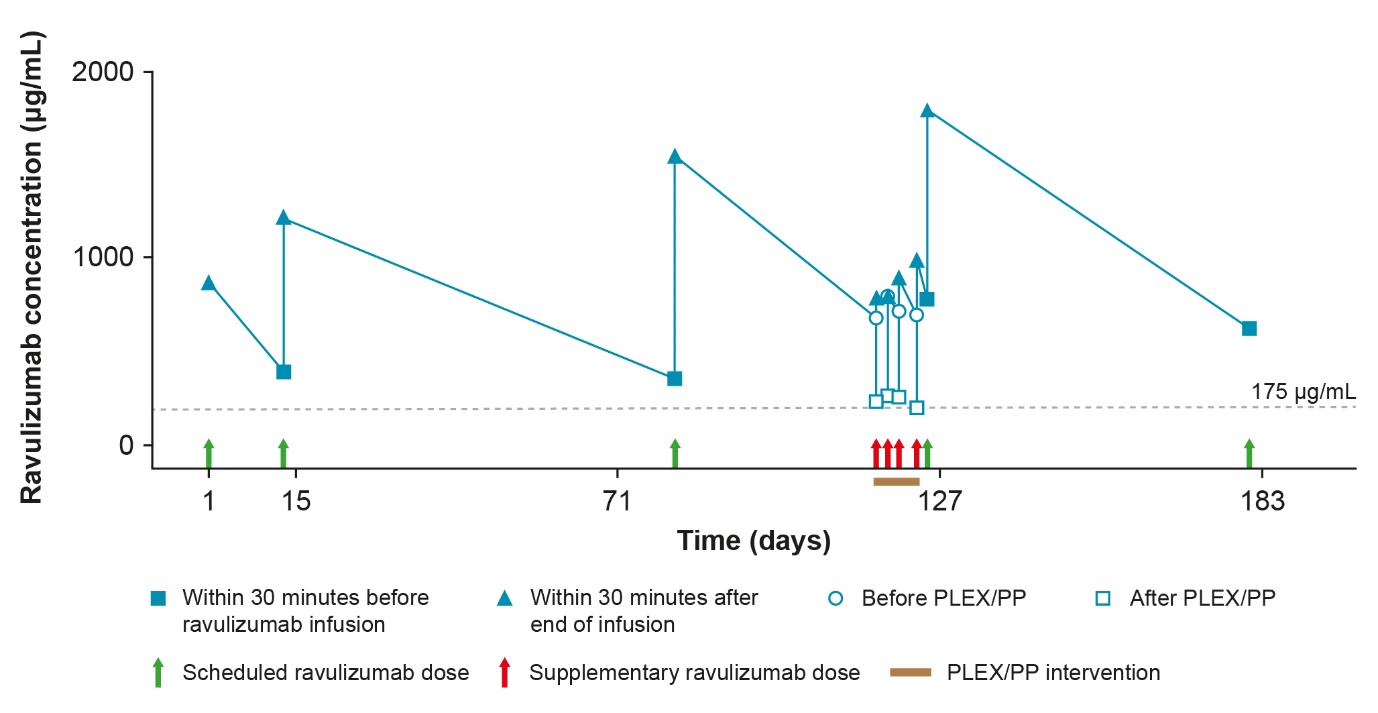


The dashed line represents the pharmacokinetic therapeutic target threshold.

PLEX/PP = plasma exchange/plasmapheresis

**Supplementary Fig. S2** Example of impact of IVIg intervention and supplemental ravulizumab doses on the concentration–time profile of ravulizumab


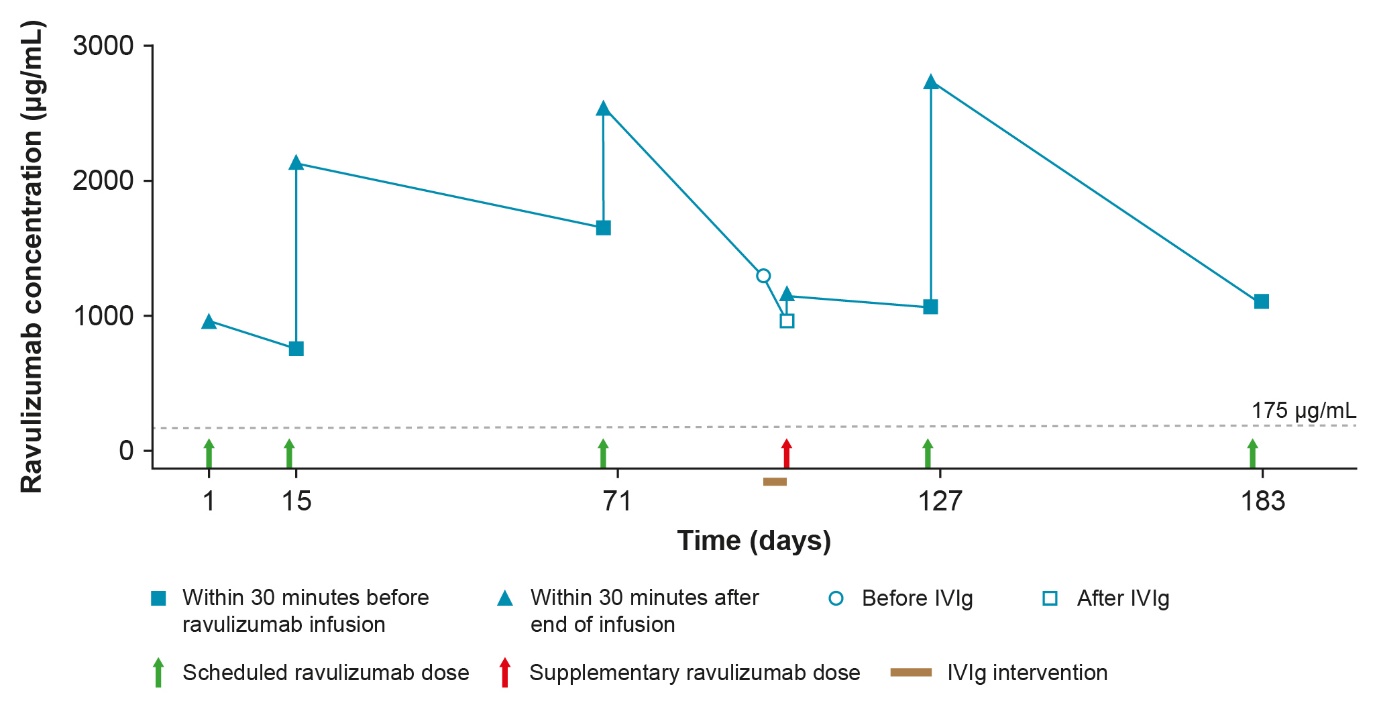


The dashed line represents the pharmacokinetic therapeutic target threshold.

IVIg = intravenous immunoglobulin

**Supplementary Fig. S3** Mean (SD) serum free C5 concentrations and mean (SD) MG-ADL total scores across 26 weeks of treatment with ravulizumab

**
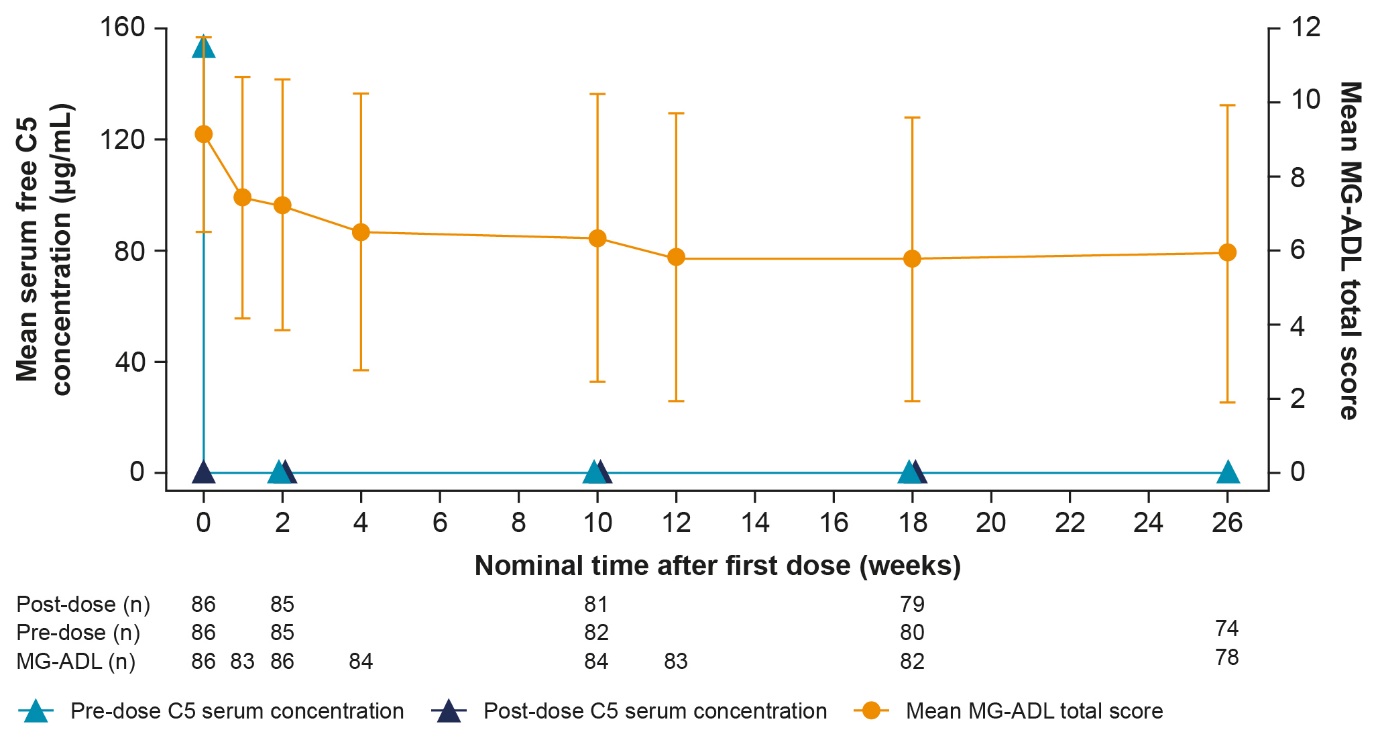
**
